# Crisis-management, Anti-stigma, and Mental Health Literacy Program for University Students (CAMPUS): A preliminary evaluation of suicide prevention

**DOI:** 10.12688/f1000research.111002.1

**Published:** 2022-05-05

**Authors:** Asumi Takahashi, Hirokazu Tachikawa, Ayumi Takayashiki, Takami Maeno, Yuki Shiratori, Asaki Matsuzaki, Tetsuaki Arai

**Affiliations:** 1 Hokusei Gakuen University, Sapporo, Hokkaido, 0048631, Japan; 2 Faculty of Medicine, University of Tsukuba, Tsukuba, Ibaraki, Japan

**Keywords:** suicide prevention, suicide prevention education, university students

## Abstract

**Background:** University students have specific risk factors for suicide, necessitating targeted prevention programs. This preliminary study evaluated the efficacy of the Crisis-management, Anti-stigma, Mental health literacy Program for University Students (CAMPUS) for reduction of risk factors and promotion of preventative behaviors.

**Methods: **A total of 136 medical students attended the CAMPUS as a required course at the national university in Japan. The CAMPUS consisted of a lecture and two group sessions covering mental health literacy, self-stigma, and gatekeeper efficacy (e.g., identifying and helping at-risk individuals). The students were asked to role-play based on a movie about gatekeepers and scripts about self-stigma and suicide-related issues. Participants completed questionnaires on suicidal thoughts, depression, help-seeking intentions, self-efficacy as gatekeepers, self-concealment, and self-acceptance. A total of 121 students completed the questionnaires pre- and post-program, and 107 students also responded six months later.

**Results:** Students demonstrated significantly reduced overall suicide thoughts six months post-program compared to before the program. In addition, gatekeeper self-efficacy, help-seeking intentions for formal resources, and self-acceptance were improved in the students six month after the program.

**Conclusions:** The CAMPUS suggested effective at reducing suicidal people and promoting preventative psychological tendencies among medial students. This study was a one-group pre post design study without control group. The CAMPUS program was delivered as a mandatory requirement to a group with relatively low suicide risk. Further studies are required to assess its suitability for the general university student population.

## Introduction

Suicide is the leading cause of death among Japanese university students, with about 350 dying by suicide each year. Between 10% and 40% of university students report thoughts of suicide, substantially higher than among the general population ( Jain *et al.*, 2012; Osama *et al.*, 2014; Peltzer *et al.*, 2017; Sun *et al.*, 2017). Risk factors for suicide among university students include poor academic performance, pre-existing mental disorders exacerbated by stress, a history of adverse childhood experiences, smoking, alcohol and drug use, and rejection by family or colleagues ( Jain *et al.*, 2012; Osama *et al.*, 2014; Zheng & Wang, 2014; Peltzer *et al.*, 2017). Many of these factors are specific to the university environment, so programs tailored to this population are required for suicide prevention.

Some universities already have shown the effectiveness of suicide prevention programs. For example, an online psychoeducational program called ProHelp was shown to improve participants’ help-seeking attitudes and suicide literacy ( Han *et al.*, 2018). An online program including multiple technological components, including a website and two social media networking applications, was also reported as useful by a wide spectrum of students ( Manning & VanDeusen, 2011). Gatekeeper training has been shown to help students identify colleagues at risk of suicide and provide help such as referral to mental health services ( Kibler & Haberyan, 2008; Indelicato *et al.*, 2011). In Japan, Katsumata *et al.* (2017) reported that a 4-h suicide prevention education program improved attitudes toward suicide and an attitude which was needed in a peer support toward self-destructive behaviors.

However, these studies did not evaluate direct effects on mental health and suicidal ideation. Harrod *et al.* (2014) reviewed eight studies on primary prevention of suicide among university students and concluded that policy interventions including means restriction lowered the suicide incidence compared to universities without such interventions ( Joffe, 2008). However, they also concluded that studies examining the effects of classroom instruction on suicidal behavior or long-term outcomes are still required. A suicide prevention education program for high school students, the Youth Aware Mental Health (YAM), was reported to reduce suicidal ideation and suicide attempts at 12 months post-program (although not after 3 months) compared to a control group ( Wasserman *et al.*, 2015). As programs may show only transient benefits or not help those at higher risk, it is important to examine the intermediate and longer-term efficacy for prevention of suicidal behaviors and promotion of preventative behaviors.

The university-targeted mental health education program Crisis management, Anti-stigma, Mental Health Literacy Program for University Students (CAMPUS) consists of a lecture, role-play sessions, and discussion modules focusing on three main components of suicide prevention: 1) mental health literacy, 2) anti-stigma, and (3) crisis management. Mental health literacy is defined by Jorm *et al.* (1997) as “knowledge and beliefs about mental disorders which aid their recognition, management or prevention.” Mental health literacy can improve mental health among college students ( Rafal *et al.*, 2018) and is positively correlated with help-seeking behavior ( Gorczynski *et al.*, 2017). The CAMPUS focuses on mental health literacy particularly pertinent to university students and adolescents, including stress, depression, suicidal thoughts, self-care, and support resources. The anti-stigma component aims to combat self-stigma and provide coping methods ( Corrigan & Watson, 2002; Goffman, 1990). Several studies have found that people with strong self-stigma regarding mental health issues tend to have low self-esteem, low self-efficacy, and low help-seeking intention ( Corrigan & Watson, 2002; Pattyn *et al.*, 2014). The CAMPUS also addresses misunderstandings about suicide and the reasons for acquiring self-stigma such as experiences of discrimination, and aims to mitigate self-stigma through psychological education and exercises. Crisis management concerns gatekeeping for suicide prevention. Students learn about risk assessment, listening to those at risk, and referral to support resources. This content is referred to as gatekeeper training and is a common component of suicide prevention programs in Japan ( Hashimoto *et al.*, 2016). Although we completed CAMPUS development in 2019, we have not yet evaluated it mid-term and longer-term efficacy.

This study aimed to assess the longitudinal efficacy of CAMPUS for reduction of suicide risk factors and promotion of preventative factors and gatekeeper functions among medical students. We speculated that this program would reduce suicidal ideation and enhance preventative psychological factors among all participants.

## Methods

### Participants

One-hundred thirty-six medical students (88 males and 48 females) at the University of Tsukuba, a national university in the Kanto region of Japan, participated in this program as part of a required course. The participants had little knowledge about psychiatry and suicide because they were second-year undergraduates of average age 19.96 years (SD = 1.39; range, 19–29 years).

### Contents of CAMPUS

The main lecture is composed of three components, mental health literacy, anti-stigma, and crisis management, each lasting about 20 min, and was created using a Power Point presentation (Microsoft Office PowerPoint 2016). A handout with “fill in the blanks” format is distributed to the students. In addition, CAMPUS includes two exercise sessions. In the first gatekeeper training session, students watch a 13-min gatekeeper training film showing student consultation scenes produced by the Ministry of Health, Labour and Welfare of Japan (2016). Next, they are divided into groups of three and perform a close listening exercise and discuss the importance of listening on outcome. This session requires about 30 min and is performed in combination with the second lecture on crisis management. In the second role-playing session, new groups of three perform two role-playing exercises in which they are assigned roles of a student with self-stigma (i.e., problems with mental illness, gender identity, and bullying) or suicide thoughts, a friend who listens to the student, and an observer. After the “student” reads a detailed scenario, he consults about his issues to the friend for a few min. Thereafter, the group discusses how the “student” can resolve these issues, how to proceed with consultation, and the mental issues faced by the “student.” One role-playing session with discussion requires about 30 min. Facilitators trained in the use of CAMPUS monitor and facilitate these sessions. Students also complete various assessment scales before and after the program as detailed below.

### Procedures

Prior to the intervention, we explained the importance of suicide prevention education for undergraduate medical students to the educational coordinator staff. It could be implemented within regular or irregular classes only when the university in Japan agreed with it. We obtained permission from the dean of the medical school to implement the suicide prevention education as a required class.

The CAMPUS was conducted using three class hours (one frame, 75 min) in July 2019, with mental health literacy and anti-stigma covered in the first hour, crisis management and gatekeeper training in the second hour, and role-play sessions in the third hour. There was the lunch break between the first and second sessions. The main lecture was conducted by one author who is a psychiatrist at the university. The first author (a clinical psychologist) and three other psychiatrists acted as facilitators.

Self-reported questionnaires were administered before the program (pre-program), immediately after the program (post-program), and six months later (follow-up) in January 2020 to assess CAMPUS efficacy. The follow-up questionnaires were distributed after another class and collected during break time. The participants had no opportunity to participate in classes related to mental health and suicide for the intervening six months.

### Measures

We used the following measures to evaluate the general efficacy of the CAMPUS.

*Suicide behaviors*

The Suicidal Behaviors Questionnaire-Revised (SBQ-R) is a 4-item self-report measure of suicidal ideation ( Osman *et al.*, 2001). The total score ranges from 3 to 18. The cut-off point for non-clinical samples is 7.

*Depression*

The Patient Health Questionnaire-9 (PHQ-9) is a 9-item self-report measure in which each item is scored from 0 (Not at all) to 3 (Nearly every day) as a measure of depression severity ( Kroenke *et al.*, 2001; Pfizer, 2014). The total score ranges from 0 to 27, and the cut-off point for non-clinical samples is 5.

*Help-seeking intentions*

The General Help-Seeking Questionnaire (GHSQ) is a measure of help-seeking intentions for various resources when facing personal and emotional problems ( Wilson *et al.*, 2005). Each item is scored on a 7-point Likert scale ranging from 1 (Extremely unlikely) to 7 (Extremely likely). The help-seeking intentions for informal resources (intimate partner, friends, parents, and other relative/family members) and for formal resources (mental health experts, help lines, doctors, and ministers or religious leaders) were analyzed separately in the present research.

*Self-efficacy as a gatekeeper*

The Gatekeeper Self-Efficacy Scale (GKSES) is a 9-item measure of confidence in gatekeeper skills ( Takahashi *et al.*, 2020). The good internal consistency (Cronbach’s α coefficient was.95) and validity were confirmed using data of 875 students. The students answered questions pertaining to gatekeeping skills including their knowledge of suicide prevention using a 7-point Likert scale from 1 (Not at all) to 7 (Extremely).

*Self-stigmatize attitude*

There is no single scale appropriate to measure self-stigma because the target differs markedly among individuals. Therefore, we used two psychological indices that are closely related to self-stigma. The first was a scale measuring self-acceptance of undesirable attributes by Tsukawaki *et al.* (2009). The scale consists of eight items such as “I accept naturally my weakness.” Each item is scored on a 5-point Likert scale from 1 (Agree) to 5 (Disagree). The second was the Japanese Self-concealment Scale (JSCS) ( Larson & Chastain, 1990; Kawano, 2000), a 12-item scale assessing the tendency to actively conceal negative and distressing personal information from others. Each item is scored on a 5-point Likert scale from 1 (Strongly disagree) to 5 (Strongly agree). Self-concealment is related to suicidality ( Hogge & Blankenship, 2020), suicidal behavior ( Friedlander *et al.*, 2012), and help-seeking attitudes for psychological professional services ( Masuda *et al.*, 2012). Positive changes on these two scales are considered indicative of self-stigma mitigation.

The SBQ-R and PHQ-9 were measured prior to intervention and at the six-month follow-up, while the GKSES, GHSQ, self-acceptance, and JSCS self-concealment scales were measured at all three time points.

### Statistical analyses

We hypothesized that participants with strong suicidal ideation at baseline would demonstrate reduced ideation as measured by the SBQ-R if the program is effective, while no such change would be observed in those with low baseline ideation. Conversely, most subjects should demonstrate improved attitudes and competence related to suicide prevention.

Changes in the scale scores were evaluated using repeated measures one-way analysis of variance (RT-ANOVA) with main factors time (pre-program, immediately post-program, and six-months post-program) with post hoc Bonferroni correction for multiple comparisons. The significance level of ANOVA was adjusted by Bonferroni’s correction to *p* < 0.0083 ( *p* < 0.05 divided by six). When the main effect for time was significant, the Bonferroni test was used for post hoc analyses. The effect size is expressed by partial *η* ^2^ ( *η*_p_^2^). SPSS ver. 25.0 was used for all analyses.

### Ethical considerations

Written informed consent was obtained from all participants before the program. All students were informed of program aims and required attendance but that the questionnaires were optional. Follow-up e-mails were sent to students if the program SBQ-R or PHQ-9 score indicated cause for concern. The e-mails stated that they could always consult the University Health Center. The study was approved by the Medical Ethics Committee of the University of Tsukuba (No. 1402-1).

## Results

### Participant flow

Among the 136 medical students attending the CAMPUS program as required, 121 (79 males and 42 females, 89.0%) completed all questionnaires before and immediately after the program. In addition, 107 students (67 males and 40 females, 78.7%) answered the follow-up questionnaires at six months post-program.

### Correlations between pre- and post-program psychometric scores

The correlations between pre- and post-program test scores are shown in Table 1. Pre-program suicidal ideation as measured by the SBQ-R was positively but weakly correlated with depression severity as measured by the PHQ-9 ( *r* = 0.25). Despite greater depression severity among participants at higher scores (showing greater suicidal ideation), SBQ-R was negatively correlated with help-seeking intention for informal resources as assessed by the GHSQ ( *r* = −0.25). Also, SBQ-R was positively correlated with greater self-concealment score ( *r* = 0.37). Poor self-acceptance was more strongly correlated with depression ( *r* = −0.34) than with suicidal ideation ( *r* = −0.19). Self-efficacy as a gatekeeper was correlated with help-seeking intentions for formal resources both pre-program ( *r* = 0.33) and post-program ( *r* = 0.39). Self-efficacy as a gatekeeper and self-acceptance were unrelated pre-program ( *r* =0.04), but significantly correlated post-program ( *r* =0.40).

**Table 1.** Correlations among psychological scale scores of CAMPUS program participants.

Scale	1. SBQ-R		2. PHQ-9		3. GHSQ (formal)		4. GHSQ (informal)		5. GKSES		6. Self-concealment		7. Self-acceptance	
2.	.25	**	-											
3.	−.25	**	−.25	**	-		.43	***	.24	**	−.21	*	.17	
4.	−.02		−.03		.35	***	-		.39	***	.10		.04	
5.	.13		−.05		.19	*	.33	***	-		−.06		.40	***
6.	.37	***	.39	***	−.24	**	.08		.11		-		−.28	**
7.	−.19	*	−.34	***	.14		.11		.04		−.29	**	-	

Note. n = 121. The lower left shows the correlation coefficients pre-program, while the upper right shows the correlation coefficients post-program.

^*^ *p* < 0.05,

^**^ *p* < 0.01,

^***^ *p* < 0.001.

### Longitudinal effects of the CAMPUS

The numbers of participants according to SBQ-R scores pre- and post-program are shown in Table 2. Among 107 participants who completed all follow-up tests, 59 scored 3 (lowest limit) on the SBQ-R pre-program while 48 scored 4 or more (44.9%). Of those, eight people had more than the cut-off points (7). At six-months follow-up, a greater number of participants scored 3 (n = 70) and fewer scored 4 or more (n = 37, (34.6%; *p* = 0.04 by McNemar’s test). Of those, six people had more than 7 cut-off points. The highest score was 9 points pre-program, but it was 11 points after six months.

**Table 2.** Distribution of SBQ-R scores before the CAMPUS program and at a 6-month follow-up.

SBQ-R score	Pre-program		Follow-up	
	*N*	%	*N*	%
3	59	55.1	70	57.9
4	30	28.0	17	14.0
5	8	7.5	11	9.1
6	2	1.9	3	2.5
7	4	3.7	2	1.7
8	3	2.8	1	0.8
9	1	0.9	2	1.7
10	0	0	0	0
11	0	0	1	0.8
*M* ( *SD*)	3.83 (1.31)		3.76 (1.44)	

Note. n = 107. SBQ-R: Suicidal Behaviors Questionnaire-Revised.

Next, average scores for all pre-program, post-program, and follow-up tests as well as the results of one-way RT-ANOVA are shown in Table 3.

**Table 3.** Average psychological scale scores and ANOVA results of CAMPUS participants.

Scale	*N*	Pre	Post	Follow-up	Main effect of time			Differences
		*M* ( *SD*)	*M* ( *SD*)	*M* ( *SD*)	*F*-value	*p*	*η _p_* ^2^	
Depression	107	3.28 (2.95)		3.12 (3.42)	0.26	.61	.00	
Help-seeking intention for informal resources	99	16.13 (4.61)	17.09 (4.51)	16.79 (4.32)	3.76	.03	.04	pre<post ^**^
Help-seeking intention for formal resources	102	10.73 (4.62)	13.43 (4.64)	11.67 (4.70)	14.88	<.001	.13	pre<post ^**^, post>follow ^**^
Gatekeeper self-efficacy	106	25.74 (10.61)	43.75 (7.48)	37.75 (11.23)	144.00	<.001	.58	pre<post ^***^, post>follow ^***^, pre<follow ^***^
Self-acceptance	107	25.07 (5.98)	26.80 (5.23)	26.22 (4.70)	7.55	<.001	.07	pre<post ^**^
Self-concealment	107	39.64 (15.04)	40.41 (13.79)	38.50 (15.09)	1.25	.29	.01	

Note. n = 107. Significance level of main effects and interaction = *p* < 0.0083. Pre = pre-program, Post = post-program, Follow-up = 6 months after the program. Post-hoc analysis

^**^ *p* < 0.01,

^***^ *p* < 0.001.

Depression scores were below the clinical cut-off point pre-program and at follow-up. The PHQ-9 depression severity scores were reduced at follow-up compared to pre-program baseline, but main effects of time were not significant ( *F*(1, 105) = 0.26, *p* = 0.61, *η*_p_^2^ = 0.00).

Help-seeking intentions for informal resources were greater in the post-program than in the pre-program, but the main effect of time was not significant ( *F*(1.68, 163.08) = 3.76, *p* = 0.03, *η*_p_^2^ = 0.04). There was a significant main effect of time on help-seeking intention for formal resources ( *F*(1.57, 157.33) = 14.88, *p* < 0.001, *η*_p_^2^ = 0.13). Scores were higher post-program, but decreased significantly at follow-up.

There was a significant main effect of time on gatekeeper self-efficacy ( *F*(2, 208) = 142.45, *p* < 0.001, *η*_p_^2^ = 0.58). Participants demonstrated higher scores post-program, but scores decreased significantly from post-program to follow-up. Nonetheless, the score at follow-up was still higher than pre-program, indicating a long-term enhancement of gatekeeper self-efficacy.

There was also a main effect of time on self-acceptance score ( *F*(2, 210) = 7.55, *p* < 0.001, *η*_p_^2^ = 0.07), and the participants demonstrated significantly improved scores post-program as well as at follow-up. Alternatively, self-concealment was not changed significantly ( *F*(1.74, 182.80) = 1.25, *p* = 0.29, *η*_p_^2^ = 0.01).

## Discussion

The purpose of this study was to examine the immediate and longer-term benefits of the CAMPUS for reducing suicidal risk factors and promoting preventative psychological factors among university students. The proportion of participants at higher SBQ-R score was significantly lower at six months post-program compared to baseline. Further, participants demonstrated improved self-acceptance and gatekeeper self-efficacy, two important suicide mitigation factors. Other suicide educational programs for university students have been shown to improve literacy and attitudes toward suicide, but have not been demonstrated to reduce suicidal ideation or behavior ( Han *et al.*, 2018; Katsumata *et al.*, 2017; Indelicato *et al.*, 2011; Kibler & Haberyan, 2008). In contrast, this relatively brief and easily delivered program tailored to the special needs of university students reduced suicidal ideation (as revealed by the SBQ-R) at follow-up. Thus, CAMPUS can be effectively implemented on a smaller scale to reduce suicidal ideation, warranting studies on broader application throughout this and other institutions.

However, a few students had higher SBQ-R scores after six months, so CAMPUS as a primary suicide prevention program was not effective for all subjects, particularly those at highest risk at baseline in this study. Other programs may share this limitation. For example, students with severe suicidal ideation and suicide attempts were immediately taken for clinical assessment and provided treatment; hence, they were excluded from the analysis of the YAM ( Wasserman *et al.*, 2015). It is thus critical to underscore that the CAMPUS is a primary preventative measure and cannot replace targeted medical interventions. In our study, the number of students with SBQ-R ≥ 7 cut-off points was small; hence, further research is needed on the effects of CAMPUS on high-risk students.

Educational contents and role-playing appeared to facilitate gatekeeper skills and self-acceptance, even after six months. Thus, the CAMPUS achieved one of its central aims, to enhance awareness of suicide risk in others, thereby increasing the likelihood that high-risk individuals are listened to and referred to mental health services.

Surprisingly, this reduction in suicidal ideation was not accompanied by marked changes in depression severity scores, possibly due to the relatively low baseline depression scores among the participants. Indeed, depression scores were significantly lower than in a previous study of medical students in Serbia ( Miletic *et al.*, 2015). However, the correlation between depression and suicide risk pre-program was weak. Mild depression may be relatively common among university students due to academic pressures and in some cases distance from family. These results suggest that the CAMPUS directly targets theme of suicide rather than theme of depression.

Importantly, CAMPUS also increased help-seeking intention for formal resources. Recognition of need and confidence in treatment benefits are important factors promoting help-seeking ( Czyz *et al.*, 2013; Downs & Eisenberg, 2012). Considering the positive correlation between help-seeking intention and gatekeeper self-efficacy, the students with high gatekeeper self-efficacy are more likely to consult formal resources for themselves and to refer others to these resources.

On the other hand, the CAMPUS did not improve self-concealment, implying that many participants were still reticent to discuss suicidal thoughts. Even though some participants role-played a suicidal person, the role-playing friends likely did not include anyone that the participant would actually consult. Those who can seek help from informal resources report greater well-being ( Goodwin *et al.*, 2016), so it is important to consult familiar people. Self-concealment may hinder help-seeking intentions for informal resources as low help-seeking intention for informal resources was associated with higher self-concealment in the present study. Surprisingly, self-concealment rating tended to increase immediately after the program. They may have found that “it was surprisingly difficult or uncomfortable to tell people about suicidal ideation” during the role-playing session, resulting in greater self-concealment. However, the variance in self-concealment scores was larger than other measures, indicating large inherent differences and responses to the role-playing exercise. Knowing a close friend or relation who has sought help can facilitate help-seeking behavior ( Disabato *et al.*, 2018), so help-seeking intention to informal resources may be improved by discussing experience of consulting close people in the CAMPUS exercises. Lindow *et al.* (2020) reported that teenage peers and school personnel did not promote help-seeking intention by YAM participants despite the program improving mental health knowledge and reducing the stigma surrounding mental illness; however, it did promote help-seeking behaviors. This suggests that students may reach out to informal resources even if they do not intend to seek help for themselves. For example, university students may seek or offer help for family members and friends in the flow of irrelevant conversation. Therefore, it is necessary to measure help-seeking behaviors among CAMPUS participants in the future.

### Limitations

This study has several limitations. First, this study was a one-group pre-post design; hence, it was not possible to randomize or establish a control group due to conflicts with university’s regular courses and educational ethical consideration. The psychological scale scores confirmed after six months should be influenced by a variety of factors that affect the individual during the six months of student life. However, applying for suicide prevention education in Japanese universities is a hurdle due to the framework in which the classes are conducted. Realistically, it would be desirable to be able to compare the results with those of the control group, preferably by means of a crossover study in which the timing of the education is shifted. Second, this study looked at the effects of self-reported selective questionnaires alone. It is necessary to examine the changes in students’ attitudes and actions, which do not appear in the questionnaire, through qualitative methods, such as interviews. Third, although the questionnaire responses were voluntary, attending the CAMPUS was compulsory. Required participation may have raised gatekeeper self-efficacy and knowledge of suicide, but coercion in education can induce psychological reactance ( Brehm, 1966). However, suicide prevention programs for elementary and junior high schools are similarly required, and required classes do not always have a negative impact on students. Nonetheless, it is necessary to consider how the form of education affects the program.

## Data availability

The raw data supporting the results of this article cannot be shared as stated by the ethical committee that approved this study. Researchers interested in accessing the data will need to require to be approved by the Ethical Committee of University of Tsukuba. Requests to access these datasets should be directed to First author, Asumi Takahashi, a-takahashi@hokusei.ac.jp.
