# Supplementary material for: Crisis-management, Anti-stigma, and Mental Health Literacy Program for University Students (CAMPUS): A preliminary evaluation of suicide prevention
Source: F1000Res. 2023 Jul 3;11:498. Originally published 2022 May 5. [Version 2] doi: 10.12688/f1000research.111002.2 (PMC10422051; doi:10.12688/f1000research.111002.2)
Supplement: Peer review history

# Peer review: Reviewer response for version 2

**DOI:** 10.5256/f1000research.151066.r183708

**Published:** 2023-08-11

**Authors:** Kate Wolitzky-Taylor

**Affiliations:** 1 University of California Los Angeles, Los Angeles, CA, USA

The revisions have improved the manuscript and are acceptable.

Is the work clearly and accurately presented and does it cite the current literature?

Yes

If applicable, is the statistical analysis and its interpretation appropriate?

Partly

Are all the source data underlying the results available to ensure full reproducibility?

Yes

Is the study design appropriate and is the work technically sound?

Yes

Are the conclusions drawn adequately supported by the results?

Yes

Are sufficient details of methods and analysis provided to allow replication by others?

Yes

Reviewer Expertise:

NA

I confirm that I have read this submission and believe that I have an appropriate level of expertise to confirm that it is of an acceptable scientific standard.

# Peer review: Reviewer response for version 2

**DOI:** 10.5256/f1000research.151066.r183707

**Published:** 2023-07-12

**Authors:** Erminia Colucci

**Affiliations:** 1 Department of Psychology, Middlesex University London, London, UK

I have reviewed and am happy to approve.

Is the work clearly and accurately presented and does it cite the current literature?

Partly

If applicable, is the statistical analysis and its interpretation appropriate?

I cannot comment. A qualified statistician is required.

Are all the source data underlying the results available to ensure full reproducibility?

Yes

Is the study design appropriate and is the work technically sound?

Partly

Are the conclusions drawn adequately supported by the results?

Yes

Are sufficient details of methods and analysis provided to allow replication by others?

Yes

Reviewer Expertise:

Suicide prevention

I confirm that I have read this submission and believe that I have an appropriate level of expertise to confirm that it is of an acceptable scientific standard.

# Peer review: Reviewer response for version 1

**DOI:** 10.5256/f1000research.122670.r161748

**Published:** 2023-02-28

**Authors:** Erminia Colucci

**Affiliations:** 1 Department of Psychology, Middlesex University London, London, UK

This is a valuable piece of work considering the high prevalence of suicidal behaviour in this population, however there are a few issues that I suggest to address:

- Please support this statement 'the participants had little knowledge about psychiatry and suicide because they were second-year undergraduates'.
- I have great concerns about labelling an intervention as 'suicide prevention' when the training materials are about mental health literacy as it is based on the (north-European/American) biomedical model of suicide that sees suicide fundamentally as caused by mental illness. This belief is contested by cultural/global mental health and critical suicidology scholars and the authors must justify their decision to develop a training based on the English speaking countries MHFA, when they also had the option, for instance, to use the tool that was specifically developed for Japan but is not even mentioned in the article, i.e. Colucci *et al.,* 2011 ^1^.
- Similarly measuring depression as distal measurement for suicide is based on the same assumption indicated above and the authors should show first of all awareness of this belief and second justify it (and paper contains many of these assumptions as demonstrated by this statement 'Surprisingly, this reduction in suicidal ideation was not accompanied by marked changes in depression severity scores').
- The limitations section should include the limitations stated above, namely that the authors used MH literacy instead of suicide literacy specifically.

Is the work clearly and accurately presented and does it cite the current literature?

Partly

If applicable, is the statistical analysis and its interpretation appropriate?

I cannot comment. A qualified statistician is required.

Are all the source data underlying the results available to ensure full reproducibility?

Yes

Is the study design appropriate and is the work technically sound?

Partly

Are the conclusions drawn adequately supported by the results?

Yes

Are sufficient details of methods and analysis provided to allow replication by others?

Yes

Reviewer Expertise:

Suicide prevention

I confirm that I have read this submission and believe that I have an appropriate level of expertise to confirm that it is of an acceptable scientific standard, however I have significant reservations, as outlined above.

## References

[1] : Mental Health First Aid guidelines for helping a suicidal person: a Delphi consensus study in Japan. *Int J Ment Health Syst* .2011;5: 10.1186/1752-4458-5-12 12 10.1186/1752-4458-5-12 21592409PMC3108941

# Response

**Published:** 2023-06-06

**Authors:** Asumi Takahashi

**Affiliations:** Hokusei Gakuen University, Japan

**Thank you for your comments and suggestions. We have underlined any additions and struck out deletions. We have responded to the reviewers' comments below. We appreciate your re-review.**

- Please support this statement 'the participants had little knowledge about psychiatry and suicide because they were second-year undergraduates'.

**Answer: We have added the information about Japanese medical students as follows:**

Most Japanese medical students enter medical school straight out of high school, so the participants were second-year undergraduates averaging 19.96 years (SD = 1.39; range, 19–29 years) in age and had little knowledge of psychiatry and suicide. In a survey of Japanese students on their understanding of suicide (Takahashi et al., 2020), the average percentage of correct answers by students in health majors such as medicine and nursing was 56.9%, which was lower than that of Australian National University students (63.4% ) (Calear, Batterham, Trias & Christensen, 2021). (in Methods-Participants)

- I have great concerns about labelling an intervention as 'suicide prevention' when the training materials are about mental health literacy as it is based on the (north-European/American) biomedical model of suicide that sees suicide fundamentally as caused by mental illness. This belief is contested by cultural/global mental health and critical suicidology scholars and the authors must justify their decision to develop a training based on the English speaking countries MHFA, when they also had the option, for instance, to use the tool that was specifically developed for Japan but is not even mentioned in the article, i.e. Colucci *et al.,* 2011 ^1^ .
- Similarly measuring depression as distal measurement for suicide is based on the same assumption indicated above and the authors should show first of all awareness of this belief and second justify it (and paper contains many of these assumptions as demonstrated by this statement 'Surprisingly, this reduction in suicidal ideation was not accompanied by marked changes in depression severity scores').

**Answer: Thank you for this insightful comment. Some parts of this program indeed treat mental illness as strongly related to suicide, and many parts are based on the medical model. On the other hand, we would like to confirm that mental health literacy is only one element of this program: The crisis-management component deals with suicide literacy, and the anti-stigma component deals with the difficulty in seeking help specific to Japanese adolescents and does not focus solely on mental health.**

**As Colucci *et al. *(2011) point out, the discourse that "talking about suicide increases anxiety" is believed in Japan, and thus education that does not even use the word "suicide" is recommended (Motohashi *et al.*, 2019) even when the goal of the teaching is suicide prevention for adolescents. In such a situation, the word "suicide" is clearly used in our program. Therefore, we believe that the fact that "suicide" is not explicitly taught about in Japan and that the purpose of this intervention is suicide prevention justifies labeling this program as suicide prevention education.**

**The elements of mental health literacy are based on YAM, for which there is evidence. This is important because the program has been validated as suicide prevention education.**

**Considering the above, the following changes have been made to support the justification for addressing mental health literacy and the fact that this intervention can be said to be suicide prevention.**

1. **Depression is a major causative factor for suicide among Japanese university students. We have added the following to the introduction:**

Although the background of student suicides in Japan is often unclear in the reports from individual universities, when combined with national suicide statistics, it is estimated that poor academic performance, worries about career paths, and mental health issues are often behind the suicides. In a large survey involving 80% of Japanese universities and colleges for the 2020-2021 biennium, 52.8% (n=662) of the deceased students reported by each university (2-year total N=1,254) were suicides or suspected suicides. The presumed history was mostly unknown (n=470), but the most common reasons tended to be related to poor academic performance (n=77), worries about career paths (n=60), feelings of isolation and loneliness (n=48), and worries about illness (n=35) (Ministry of Education, Culture, Sports, Science and Technology of Japan, 2021; 2022). National suicide statistics also show that male students are more likely to suffer from poor academic performance (18.4%), worries about career paths other than entrance exams (15.3%), and depression (10.9%). In comparison, female college students are more likely to suffer from depression (21.7%), worries about career paths (12.0%), and poor academic performance (10.1%) (Ministry of Health, Labour and Welfare of Japan, 2022). In accordance with the WHO (2014) report that mental illness is likely to be behind suicides, universities are required to implement preventative mental health care and to detect students with mental health concerns early. (in Introduction)

**1. We added (to the introduction) that the program incorporates an element of anti-stigma by acknowledging that Japanese students find it difficult to seek help.**

Only 10-20% of students who died by suicide in Japan were connected to on-campus resources such as student counseling (Ministry of Education, Culture, Sports, Science and Technology, 2021; 2022). Therefore, there is a need to specifically address the stigma of mental health issues and suicide and facilitate help-seeking behavior. (in Introduction)

**2. We have also added a comment regarding YAM, for which there is ample evidence.**

CAMPUS began development in 2017 and referred to YAM, an evidence-based education program. (in Introduction)

**3. We have added a comment that Japan is challenged to explicitly use the WORD "suicide" in a culture that typically avoids it.**

One of the features of CAMPUS is its explicit treatment of the word "suicide." As noted by Colucci *et al.* (2011), even experts in Japan acknowledge that there is more avoidance of the topic of suicide in Japan than in other countries. Perhaps for this reason, suicide prevention education in Japan is encompassed by "education on how to give SOS" and "stress management education," and the terms suicide and suicide prevention are not actively used (Motohashi *et al.*, 2019). CAMPUS, however, has at its core, a fearless dialogue around suicide. (in Introduction)

**4. We have added the following to the Methods section to indicate that the lesson is positioned at the beginning of the education program as a story about suicide.**

At the beginning of the class, it was clearly explained that the class was about suicide and positioned as a lesson that is part of suicide prevention. (in Method - Contents of CAMPUS)

**5. We have added a note about the issues raised in the scenario to indicate that the content covered in the role play is diverse.**

 After the “student” reads a detailed scenario, they consult with their friends about the issues for a few min. The background information leading to the issue of suicide is set in the context of poor academic performance, career path concerns, relationships among college students, and so on. (in Method - Contents of CAMPUS)

- The limitations section should include the limitations stated above, namely that the authors used MH literacy instead of suicide literacy specifically. 

**Answer: We have added to the limitations section as follows:**

Finally, CAMPUS was more concerned with mental health literacy than suicide literacy, which might have resulted in a possible lack of information about suicide. When aiming for direct prevention of suicide rather than mental health issues, one approach would be to address the difficulties of student life, such as studying and finances, that underlie student suicide or to address their knowledge of suicide, such as how to limit the means of suicide (cf. lethal means counseling) and safety planning. It remains to be seen what theoretical model should be followed in structuring the content that can educate students in a limited time. (in Limitations)

# Peer review: Reviewer response for version 1

**DOI:** 10.5256/f1000research.122670.r161741

**Published:** 2023-02-17

**Authors:** Kate Wolitzky-Taylor

**Affiliations:** 1 University of California Los Angeles, Los Angeles, CA, USA

This paper presents data from a gatekeeper program in Japan with undergraduate medical students. There are many strengths of the paper, including that it is well-written, with clear methodology and presentation of results. It also includes several key outcome variables, including suicide risk, depression, and changes in stigma, attitudes, and awareness, encompassing many of the critical outcomes in this type of work. Other prior studies have not examined all of these interesting outcomes. However, there are a few minor issues that could strengthen the paper.

 First, for an international audience, it may be helpful to describe the “undergraduate medical student” sample, as medical school is a professional school that comes after an undergraduate education in many Western countries.

 Second, there are some minor concerns about the interpretation of the results that should be addressed. Specifically, the changes in the suicide measure are presented in a way that is either confusing or may distort the magnitude of the change. I would suggest reporting a dichotomous cutoff of high risk v low risk, in which the authors put people into two categories pre-intervention and follow-up, and then do a chi-square test so there can be an actual inferential statistic there. Also, the description of the depression scores improving though non-statistically significantly should be changed to “no significant improvement” – the change was very small and the effect size was 0, so it is misleading to say there was any change.

 Otherwise, I think this is a nice paper that makes a contribution to the literature.

Is the work clearly and accurately presented and does it cite the current literature?

Yes

If applicable, is the statistical analysis and its interpretation appropriate?

Partly

Are all the source data underlying the results available to ensure full reproducibility?

Yes

Is the study design appropriate and is the work technically sound?

Yes

Are the conclusions drawn adequately supported by the results?

Yes

Are sufficient details of methods and analysis provided to allow replication by others?

Yes

Reviewer Expertise:

NA

I confirm that I have read this submission and believe that I have an appropriate level of expertise to confirm that it is of an acceptable scientific standard, however I have significant reservations, as outlined above.

# Response

**Published:** 2023-06-06

**Authors:** Asumi Takahashi

**Affiliations:** Hokusei Gakuen University, Japan

**Thank you for providing these insights. We have responded to the reviewers' comments below. In addition, we have underlined any additions and have struck out any deletions. We hope that the manuscript will now be ready for indexing.**

- First, for an international audience, it may be helpful to describe the “undergraduate medical student” sample, as medical school is a professional school that comes after an undergraduate education in many Western countries.

**Answer: Thank you for bringing this to our attention. We have added the information about Japanese medical students as follows:**

Most Japanese medical students enter medical school straight out of high school, so the participants were second-year undergraduates averaging 19.96 years (SD = 1.39; range, 19–29 years) in age and had little knowledge of psychiatry and suicide. In a survey of Japanese students on their understanding of suicide (Takahashi *et al.*, 2020), the average percentage of correct answers by students in health majors such as medicine and nursing was 56.9%, which was lower than that of Australian National University students (63.4% ) (Calear, Batterham, Trias & Christensen, 2021). (in Methods - Participants)

- Second, there are some minor concerns about the interpretation of the results that should be addressed. Specifically, the changes in the suicide measure are presented in a way that is either confusing or may distort the magnitude of the change. I would suggest reporting a dichotomous cutoff of high risk v low risk, in which the authors put people into two categories pre-intervention and follow-up, and then do a chi-square test so there can be an actual inferential statistic there.

**Answer: Thank you for the suggestions. We have added the results of the chi-square test to ensure that the effect on high-risk students is not misinterpreted as follows:**

A chi-square test with Yates’ continuity correction before and six months after the program, dividing the participants into two groups by the cutoff score, showed no significant difference (x2(1)=0.076, n.s.). (in Results - Longitudinal effects of the CAMPUS)

 However, a few students had higher SBQ-R scores after six months and no decrease in students above the cutoff score, so CAMPUS as a primary suicide prevention program was not effective for all participants, particularly those at highest risk at baseline in this study.

 (in Discussion)

- Also, the description of the depression scores improving though non-statistically significantly should be changed to “no significant improvement” – the change was very small and the effect size was 0, so it is misleading to say there was any change.

**Answer: Thank you, we agree with your assessment and have changed the sentence describing the depression scores as follows:**

 Depression scores were below the clinical cutoff point pre-program and at follow-up. The main effects of time were not significant ( *F*(1, 105) = 0.26, *p* = 0.61, η _p_^2^ = 0.00), and depression severity showed no significant improvement. (in Results - Longitudinal effects of the CAMPUS)
